# Calpain 3 and CaMKII**β** signaling are required to induce HSP70 necessary for adaptive muscle growth after atrophy

**DOI:** 10.1093/hmg/ddy071

**Published:** 2018-03-08

**Authors:** Irina Kramerova, Jorge A Torres, Ascia Eskin, Stanley F Nelson, Melissa J Spencer

**Affiliations:** 1 Department of Neurology, David Geffen School of Medicine, University of California, Los Angeles, CA 90095, USA; 2 Center for Duchenne Muscular Dystrophy, University of California, Los Angeles, CA 90095, USA; 3 Department of Human Genetics, David Geffen School of Medicine, University of California, Los Angeles, CA 90095, USA

## Abstract

Mutations in *CAPN3* cause autosomal recessive limb girdle muscular dystrophy 2A. Calpain 3 (CAPN3) is a calcium dependent protease residing in the myofibrillar, cytosolic and triad fractions of skeletal muscle. At the triad, it colocalizes with calcium calmodulin kinase IIβ (CaMKIIβ). CAPN3 knock out mice (C3KO) show reduced triad integrity and blunted CaMKIIβ signaling, which correlates with impaired transcriptional activation of myofibrillar and oxidative metabolism genes in response to running exercise. These data suggest a role for CAPN3 and CaMKIIβ in gene regulation that takes place during adaptation to endurance exercise. To assess whether CAPN3- CaMKIIβ signaling influences skeletal muscle remodeling in other contexts, we subjected C3KO and wild type mice to hindlimb unloading and reloading and assessed CaMKIIβ signaling and gene expression by RNA-sequencing. After induced atrophy followed by 4 days of reloading, both CaMKIIβ activation and expression of inflammatory and cellular stress genes were increased. C3KO muscles failed to activate CaMKIIβ signaling, did not activate the same pattern of gene expression and demonstrated impaired growth at 4 days of reloading. Moreover, C3KO muscles failed to activate inducible HSP70, which was previously shown to be indispensible for the inflammatory response needed to promote muscle recovery. Likewise, C3KO showed diminished immune cell infiltration and decreased expression of pro-myogenic genes. These data support a role for CaMKIIβ signaling in induction of HSP70 and promotion of the inflammatory response during muscle growth and remodeling that occurs after atrophy, suggesting that CaMKIIβ regulates remodeling in multiple contexts: endurance exercise and growth after atrophy.

## Introduction

Limb girdle muscular dystrophy 2A (LGMD2A) is an autosomal recessive, progressive muscle-wasting disorder and is one of the most prevalent forms of LGMD, accounting for ∼30% of all LGMDs (1). LGMD2A is caused by mutations in the *CAPN3* gene, which encodes a non-lysosomal Ca^2+^-dependent proteinase named calpain 3 (CAPN3) (2). CAPN3 is a member of a larger family of calcium dependent proteases, but CAPN3 is unique in that it is primarily restricted to skeletal muscle (3). At this time, CAPN3’s role in muscle and the underlying reason for why loss of function mutations cause LGMD2A is still not well understood. CAPN3 deficient muscles preserve normal sarcolemmal integrity even following a period of exercise (4,5). The strong sarcolemmal integrity in this disease distinguishes it from the dystrophinopathies and suggests an altogether different pathomechanism of disease.

We previously generated a *Capn* knock out mouse (C3KO) (6), that replicates several features of LGMD2A including small fiber diameter and abnormal mitochondria (7). C3KO mice fail to re-gain muscle mass after atrophy and fail to remodel after exercise training (4,8). In addition, their muscles do not activate genes involved in the slow program, including slow twitch myofibrillar and oxidative metabolism genes (8,9). These studies suggest that CAPN3 is involved in re-growth after atrophy or a switch to the slow program after exercise training, thus establishing a role for CAPN3 in post-natal adaptive muscle growth and remodeling.

CAPN3 has been biochemically localized to multiple subcellular compartments of skeletal muscle, suggesting that it may play different roles at each subcellular location. In the sarcomere, CAPN3 interacts with the myofibrillar protein titin at two different sites: the N2-line and M-line (10). Ojima *et al.* (11) showed that CAPN3 undergoes dynamic changes in its distribution upon sarcomere extension and that this movement requires protease activity. These studies support the hypothesis that sarcomere-associated CAPN3 may act as a sensor of mechanical stretch and are consistent with the hypothesis that CAPN3 plays a role in muscle adaptation.

In addition to its location in the myofibrillar fraction, CAPN3 is also present in the cytosolic and membrane fractions of skeletal muscle (12,13). At the membrane, CAPN3 co-localizes with the triad-associated protein complex, which is the site of Ca^2+^ release to initiate muscle contraction (aka excitation–contraction coupling). CAPN3 association with triads appears to occur through its interaction with the ryanodine receptor (RyR1), the calcium release channel located in the sarcoplasmic reticulum membrane (12). In the absence of CAPN3, levels of RyR1 and other triad proteins (dihydropyridine receptor α subunit, aldolase, CaMKIIβ and others) are reduced, suggesting that CAPN3 plays a structural role in maintaining the integrity of this triad complex. Importantly, decreased concentrations of CAPN3 protein in LGMD2A patient biopsies are also accompanied by reduced levels of RyR1 (9). Consistent with the observed reduction in RyR1 protein levels, we found that Ca^2+^ fluxes elicited by single action potentials or by tetanic stimulation were significantly reduced in C3KO muscles (12,14). These results identified a novel role for CAPN3 in maintenance of the triad-associated protein complex and muscle Ca^2+^ handling.

Ca^2+^-mediated signaling plays an important role in muscle adaptation (15). The reduction in Ca^2+^ release that we observed in C3KO muscles led us to survey Ca^2+^-mediated signaling pathways, leading to the observation that CaMKIIβ-mediated signaling is blunted in C3KO mice subjected to treadmill running (8,9). Moreover, we demonstrated that failure to activate CaMKIIβ signaling after exercise is a specific feature of C3KO muscles. In contrast, when dystrophic mdx mice (a model for Duchenne Muscular Dystrophy) were subjected to a similar running protocol (8), their muscles properly activated CaMKIIβ signaling, although they exhibit some maladaptation to exercise (16,17).

CaMKII is a calcium calmodulin-dependent, serine/threonine kinase that has been previously shown to influence gene transcription in a number of different tissues (18,19). CaMKII mediates its effects through a variety of downstream signaling pathways that ultimately regulate gene transcription in the nucleus, depending on the external cues. In skeletal muscle, CaMKII has been associated with phosphorylation of histone deacetylases (HDACs), leading to their export from the nucleus (20). Removal of HDAC repression on chromatin permits MEF2-mediated transcription of slow phenotype genes. Consistent with impaired CAMKIIβ signaling in C3KO muscles, we observed a reduction in the slow fiber phenotype (8,9). Moreover, C3KO mice fail to up-regulate specific gene sets that are normally induced in remodeling following endurance exercise. Specifically, exercised C3KO mice failed to activate myofibrillar and lipid metabolism genes. In addition, we showed that running exercise activates CaMKII-p38-PGC1a (peroxisome proliferator-activated receptor gamma coactivator 1-alpha) signaling in wild type (WT) but not in C3KO mice; this signaling defect likely leads to the observed impairment in metabolic gene expression. Thus, CAPN3 and CaMKII regulate downstream signaling that induces adaptive gene expression following endurance exercise. The inability to properly activate CaMKIIβ and downstream gene expression programs that facilitate muscle adaptation to exercise may underlie LGMD2A.

Since our data showed a role for CaMKIIβ in skeletal muscle adaptation after running exercise, we sought to determine whether this pathway might facilitate muscle adaptation in other contexts, particularly in the context of muscle re-growth that occurs after a bout of atrophy. Previous studies in the C3KO mouse demonstrated insufficient and delayed growth after a bout of muscle atrophy, despite proper activation of the insulin-like growth factor-RAC-alpha serine/threonine-protein kinase (IGF-Akt) signaling pathway, a principal regulator of adult muscle growth (4). In this study, we tested this hypothesis using a model of muscle adaptation, called hindlimb suspension/reloading (HS/RL) in which adult muscle growth is stimulated by reloading after a period of disuse. This loading-dependent muscle growth has been shown to entail three major processes; (i) stimulation of protein synthesis; (ii) suppression of protein degradation and (iii) activation of muscle precursor cells (MPCs) that facilitate regeneration and growth. In addition, it had been shown that hypertrophic signals trigger induction of a cellular stress response, in particular, expression of HSPs that appear to be vital for muscle growth (21,22).

In these studies we showed that the absence of CAPN3 and reduction of CaMKIIβ activation impact adaptive gene expression during muscle growth after atrophy. Thus, these studies suggest that CaMKIIβ acts as an upstream signal to orchestrate muscle adaptation in response to different external cues.

## Results

### Attenuated muscle growth after disuse in C3KO mice

**Figure 1.**
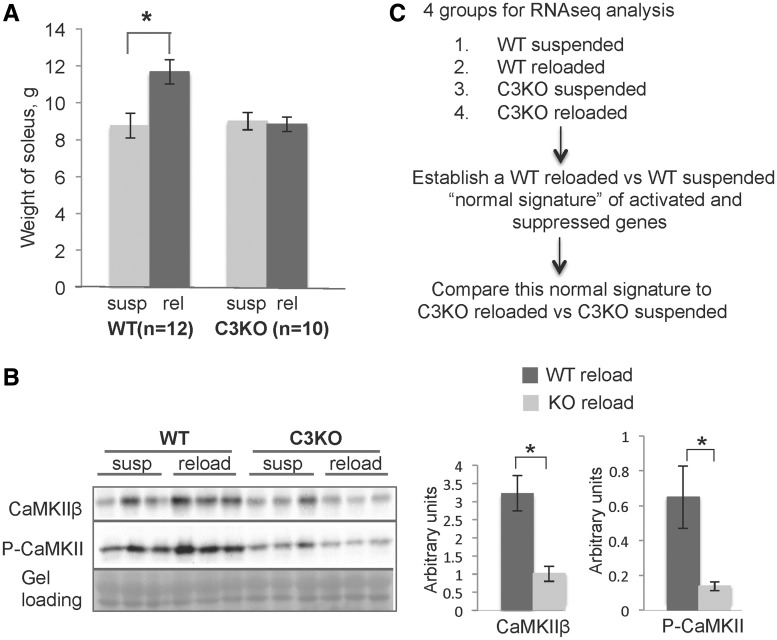
** **Muscle growth after a period of disuse was attenuated in C3KO mice. **(A)** Weight of the soleus muscles in WT and C3KO mice at the end of the suspension period (susp) and after 4 days of reloading (reload). **(B)** CaMKII was activated in response to muscle reloading in WT but not in C3KO muscles. Western blot analysis of CaMKIIβ and activated (P- Thr286) CaMKII in suspended versus reloaded muscles of WT and C3KO muscles. Quantitative analysis of the western blot is shown to the right. Asterisks indicate statistical significance with *P* < 0.05 considered statistically significant. Vertical bars represent standard deviation, *n* = 3 for each group. **(C)** An outline of the RNA-seq experiment and data analysis.

The CAPN3**-**CaMKIIβ axis was previously shown to play a role in muscle adaptation to running exercise (8,9). To examine the role of this relationship in a second context of muscle remodeling, we subjected WT and C3KO mice to HS/RL. Previous studies by our group and others have shown that HS leads to a decrease in soleus weight by ∼40% after 10 days of suspension (23). Once mice return to normal cage activity, muscle growth increases significantly between 2 and 4 days of RL and muscle mass is restored within 10–14 days (24). Previously we showed that the average cross-sectional area increased by 43.1% after 4 days of reloading in C57 BL/6 mice, whereas C3KO muscles only demonstrated an increase of 6.1% (4). In this study, these findings were reproduced. WT soleus muscle weight was increased by 33% after 4 days of RL while C3KO soleus weight did not change significantly (Fig. 1A); thus, our studies confirmed that CAPN3 deficient muscles consistently demonstrate adaptation failure in at least two different contexts of remodeling: running and reloading after atrophy.

We next asked whether the observed growth failure in C3KO mice was associated with impaired activation of CaMKIIβ. Both CaMKIIβ and activated (phosphorylated) CAMKII were increased in WT reloaded solei, suggesting that CaMKII-mediated signaling may be a universal signal that is important for remodeling in a number of contexts (Fig. 1B). C3KO solei failed to increase CaMKIIβ levels in response to muscle reloading, implicating failed CaMKIIβ signaling in attenuated muscle growth after atrophy in C3KO.

### Comparison of genes differentially regulated in response to running exercise or muscle reloading

**Figure 2.**
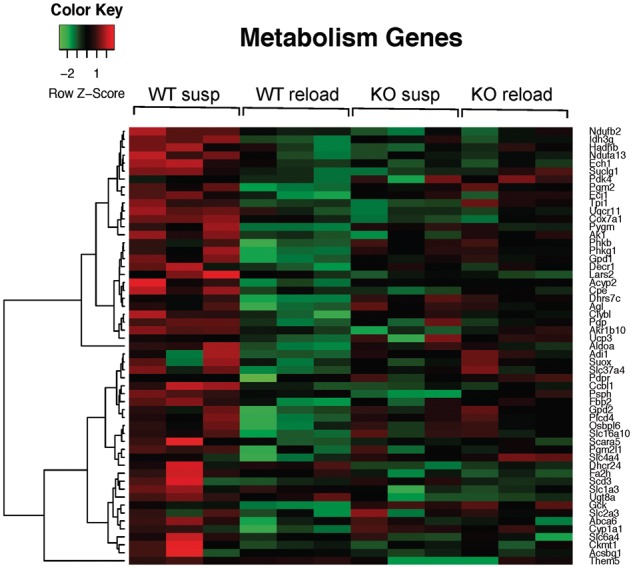
** **Downregulation of oxidative metabolism genes in WT but not C3KO muscles after 4 days of reloading. Shown is a heat map diagram of the expression levels (identified by RNA-seq) of oxidative metabolism genes in WT and C3KO muscles at the end of the suspension period (susp) or after 4 days of reloading (reload) (*n* = 6 for each group of mice).

In our prior studies we showed that blunted CaMKIIβ signaling in C3KO mice correlated with failure to activate an adaptive gene signature in response to running exercise (8). To determine whether failure to properly activate CaMKIIβ also correlated with inadequate activation of genes involved in adaptive muscle growth, we assessed gene expression patterns by RNA-seq in WT and C3KO solei subjected to HS/RL. RNA-seq is a sensitive and unbiased method of assessing and comparing transcriptomes (25). We first identified a set of genes that were differentially expressed between WT HS and WT RL muscles (i.e. a ‘normal gene signature’) and then compared this set of genes to those activated in C3KO HS versus C3KO RL muscles, to identify genes that were aberrantly expressed in the absence of CAPN3. An outline of the experiment is shown in Figure 1C. As shown in Table 1, some commonly observed gene clusters were upregulated under conditions of running or reloading, including genes involved in actin cytoskeletal rearrangements, cell adhesion and migration, as well as genes encoding extracellular matrix (ECM) and myofibrillar proteins. The most significant difference revealed by these comparisons was the expression pattern of metabolic genes, which were significantly upregulated in WT muscles in response to running exercise [(8) and Supplementary Material, Fig. S1]. In contrast, genes involved in oxidative and lipid metabolism were significantly downregulated in WT muscles after reloading (Fig. 2). Interestingly, C3KO muscles failed to regulate the same set of genes induced in WT muscles in both running and HS/RL, suggesting that failure of C3KO muscles to sense and respond appropriately to loading cues occurs in at least two settings of muscle adaptation. These studies demonstrate that an aberrant transcriptional response during remodeling is a common feature of CAPN3 deficiency.

**Table 1.** Differentially expressed genes in WT exercised versus reloaded muscles

Gene category	5 days running	4 days reloading
Myofibrillar	⇑ 22.9%	⇑ 2.4%
ECM, adhesion, migration	⇑ 22.9%	⇑ 17.8%
Actin cytoskeleton	⇑ 6%	⇑ 12%
Stress response, chaperons	⇑ 3.6%	⇑ 2.4%
Signaling, receptors	ND	⇓ 15.6%
Signaling, transcription	⇓ 36.2%	ND
Mitochindrial, oxidativeand lipid metabolism	⇑ 30.1%	⇓ 67.9%
Immune response	ND	⇑ 27.9%
Cell proliferation and growth	ND	⇑ 14.9%
Angiogenesis	ND	⇑ 3.4%
Growth and development	⇓ 48.9%	ND

### Reduced immune response to muscle reloading in C3KO mice

**Figure 3.**
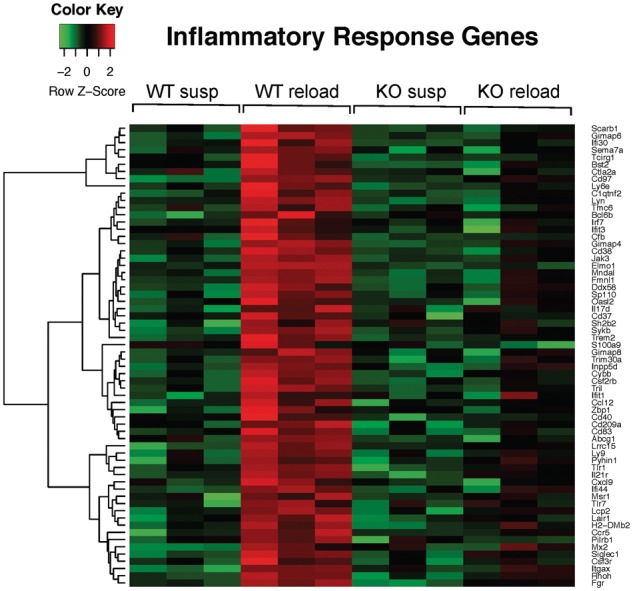
** **Upregulation of inflammatory response genes in WT but not C3KO reloaded muscles. Shown is a heat map diagram of expression levels of immune response genes in WT and C3KO muscles at the end of the suspension period (susp) or after 4 days of reloading (reload) (*n* = 6 for each group of mice).

**Figure 4.**
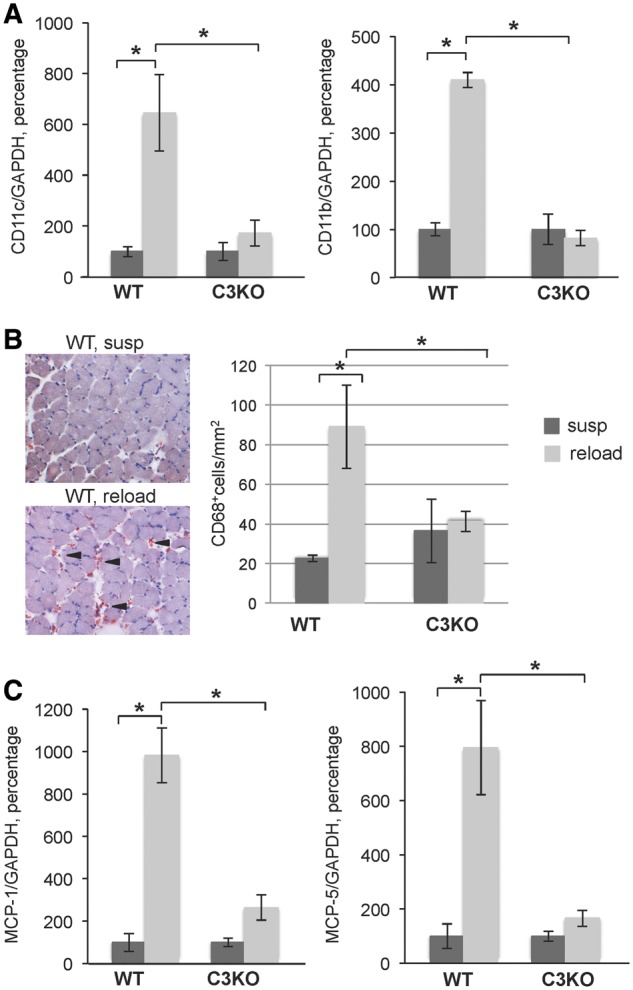
** **Attenuated leukocyte influx into C3KO muscles after 4 days of reloading. **(A)** RT-PCR validation of expression levels of the two common leukocyte surface markers CD11b and CD11c encoded by *itgam* and *itgax* genes, respectively. **(B)** Representative micrographs of cross sections from WT suspended (susp) and reloaded (reload) muscles stained for macrophage marker CD68 (left panel); quantitative analysis of CD68+ cells on WT and C3KO muscle sections (right panel). **(C)** RT-PCR validation of expression levels of MCP-1 and its homolog MCP-5 encoded by *Ccl2* and *Ccl12* genes, respectively. In each graph of RT-PCR validation, the expression level of the reloaded (reload) group is graphed relative to the suspended group (susp); the average expression of each gene in the suspended group was set at 100% for each genotype. Asterisks indicate statistical significance with *P* < 0.05 considered statistically significant. Vertical bars represent standard deviation, *n* = 3 for each group.

Muscle reloading is accompanied by a burst of intramuscular infiltration that predominantly consists of myeloid cells (macrophages, neutrophils and eosinophils). These cells play an important role in promoting muscle growth and recovery after a period of disuse (26,27). We observed that genes involved in the innate immune response represented the most abundant set of upregulated genes in WT RL muscle; however, this coordinated response was absent in C3KO muscles (Table 1 and Fig. 3). Specifically, we found that genes expressed by monocyte/macrophages and granulocytes were decreased in C3KO RL muscles compared with WT RL muscles (Table 2). RT-PCR confirmed the RNA-seq results and demonstrated that expression of the two common myeloid cell markers, CD11b and CD11c, was not induced by RL in C3KO muscles. Furthermore, the overall expression of these markers was significantly lower in C3KO RL muscles compared to WT RL muscles (Fig. 4A). In agreement with these gene expression results, C3KO RL muscles showed a reduced number of CD68-positive macrophages on tissue sections (Fig. 4B). These studies demonstrate that C3KO muscles fail to mount a proper immune response during muscle reloading.

**Table 2.** CD markers expressed by cells involved in innate immunity (monocytes/marcrophages and granulocytes); RNA-seq data

Gene	WT susp, average	WT reload, average	KO susp, average	KO reload, average	WT reload/KO reload	*P*-value, reload*
Ptprc(Cd45)	0.52	1.66	0.37	0.80	**2.08**	**0.006**
Itgam (CD11b)	0.77	2.96	0.48	1.31	**2.02**	**0.012**
Itgax (CD11c)	0.07	0.42	0.14	0.21	**2**	**0.029**
Cd14	1.69	3.77	0.80	1.72	**2.19**	**0.036**
Cd33	0.35	1.05	0.21	0.54	**1.94**	**0.023**
Cd37	1.53	3.64	1.13	1.49	**2.44**	**0.033**
Cd38	1.69	4.27	1.49	1.64	**2.6**	**0.011**
Cd40	0.66	1.84	0.38	0.54	**3.4**	**0.117**
Cd44	1.38	8.65	1.15	4.79	**1.81**	**0.061**
Cd48	1.25	2.75	0.54	1.38	**1.99**	**0.013**
Cd52	4.28	15.00	1.52	3.61	**4.16**	**0.020**
Cd53	0.78	2.67	0.44	1.32	**2.02**	**0.002**
Cd63	145.17	243.37	120.02	153.92	**1.58**	**0.045**
Cd74	54.19	126.47	26.12	65.26	**1.94**	**0.022**
Cd81	54.79	110.08	47.51	71.65	**1.53**	**0.016**
Cd84	0.47	2.14	0.31	0.94	**2.28**	**0.012**
Cd86	0.56	1.62	0.20	0.53	**3.06**	**0.002**
Cd93	4.24	15.68	4.12	7.22	**2.17**	**0.008**
Cd97	9.10	23.04	11.12	15.34	**1.5**	**0.001**
Cd209d	2.22	3.35	1.39	1.54	**2.18**	**0.031**

* Student’s test was used to calculated *P* values of WT reloaded versus KO reloaded (*n* = 6 for each group of mice).

### Decreased expression of myogenic factors in C3KO muscles

**Figure 5.**
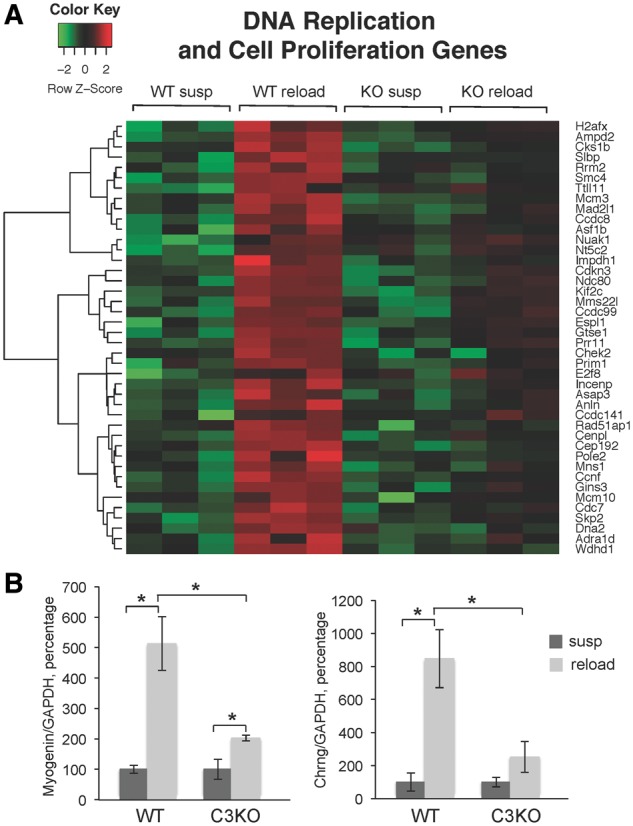
** **Decreased expression of cell proliferation and myogenic differentiation genes in C3KO reloaded muscles. **(A)** A heatmap diagram of the expression levels (identified by RNA-seq) of DNA replication and cell proliferation genes in WT and C3KO muscles at the end of the suspension period (susp) or after 4 days of reloading (reload) (*n* = 6 for each group of mice) **(B)** RT-PCR validation of expression levels of a key myogenic transcription factor myogenin and its target gene *Chrng* coding for the gamma subunit of the acetyl choline receptor. In each graph of RT-PCR validation, the expression level of the reloaded (reload) group is graphed relative to the suspended group (susp); the average expression of each gene in the suspended group was set at 100% for each genotype. Asterisks indicate statistical significance with *P* < 0.05 considered statistically significant. Vertical bars represent standard deviation, *n* = 3 for each group.

Macrophages secrete cytokines and growth factors that directly influence the proliferation and differentiation of MPCs. In experimental models of muscle injury, interference with normal immune cell influx leads to a delay in muscle regeneration [reviewed in (28)]. Our RNA-seq analysis revealed decreased expression of several chemokines in C3KO mice, particularly the CC family chemokine monocyte chemotactic protein 1 (MCP-1) and its structural homolog MCP-5. RT-PCR confirmed that MCP-1 and MCP-5 were significantly decreased in C3KO RL muscle compared to WT RL muscle (Fig. 4C). In agreement with the proposed roles for the CC chemokines in controlling cell proliferation (29), RNA-seq data demonstrated that a set of genes involved in DNA replication and cell proliferation was significantly up-regulated in WT mice but not in C3KO mice upon muscle reloading (Fig. 5A).

Analysis of the total muscle transcriptome reflects changes in all cell types present, including MPCs, fibroblasts and immune cells. Thus, the differential expression of genes involved in DNA replication and proliferation presented in Figure 5A may reflect changes in proliferation of several cell types. Since MCP-1 was shown to directly regulate proliferation of MPCs (29), we next examined whether decreased levels of MCP-1 correlated with decreased expression of the markers of myogenesis in C3KO muscles. Indeed, we found that expression levels of the transcription factor myogenin, a key marker of myogenic differentiation (30), as well as a direct target of myogenin, *Chrng* (a marker of terminal myogenic differentiation), were both significantly reduced in C3KO reloaded muscles compared to WT reloaded muscles, by RNA-seq and RT-PCR analysis (Fig. 5B). Collectively, these data suggest that in the absence of CAPN3 and an attenuated immune response, proliferation and differentiation of MPCs is reduced.

### Hsp70 is not upregulated in C3KO muscles under mechanical stress conditions

Damage-associated molecular patterns deliver signals that drive systemic responses to injury or stress, such as inflammation. One such molecule is high-mobility group box 1 (HMGB1) or alarmin. Intracellular HMGB1 can act as a DNA binding protein that modulates transcription, or HMGB1 can also be secreted by muscle and act as a pro-inflammatory cytokine (31). We assessed HMGB1 expression in our RNA-seq data and found that its expression was similar in WT and C3KO muscle (Supplementary Material, Fig. S2).

The other molecule implicated in activation of the innate immune response is inducible heat shock protein 70 (HSP70). HSP70 not only acts as an intracellular molecular chaperone that protects against cellular damage but it can also be secreted and act as a DAMP in response to various stressful conditions, including experimentally induced injury and exercise (32). HSP70 can activate both macrophages and neutrophils (33,34). Moreover, *in vivo* studies demonstrated that genetic ablation of *hsp70* attenuates the inflammatory response to muscle injury or reloading and severely impairs muscle regeneration (35).

**Figure 6.**
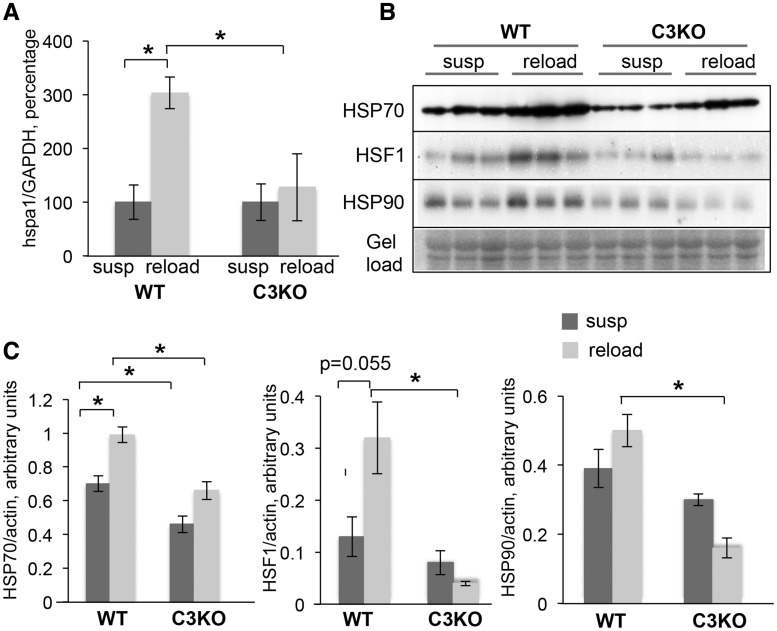
** **Inducible HSP70 was not appropriately unregulated in C3KO muscles in response to reloading. **(A)** RT-PCR validation of expression levels of *hspa1a* gene encoding inducible HSP70, in suspended (susp) and reloaded (reload) muscles. The expression level of the reloaded (reload) group is graphed relative to the suspended group (susp); the average expression of *hspa1a* gene in the suspended group was set at 100% for each genotype. **(B)** Western Blot analysis of the expression levels of HSP70, HSF-1 and HSP90 in suspended (susp) and reloaded (reload) muscles of WT and C3KO muscles. **(C)** Quantitative analysis of the western blots shown in (B). Asterisks indicate statistical significance with *P* < 0.05 considered statistically significant. Vertical bars represent SD, n = 3 for each group.

We examined our RNA-seq data to determine if HSP70 was differentially expressed between WT and C3KO muscles. HSP70 is encoded by two genes, *hspa1a* and *hspa1b*, whose products differ by only two amino acids and are believed to be functionally interchangeable proteins (collectively referred to as HSP70). These genes were found to be differentially regulated in C3KO compared to WT RL muscles (Supplementary Material, Fig. S3A). RT-PCR validation and western blot analysis confirmed that C3KO muscles had lower levels of HSP70 in the HS condition and that C3KO muscles failed to upregulate HSP70 expression to the same extent as WT muscles during RL (Fig. 6A and B, upper panel). Of note, we observed differential expression of several other *hsp* genes between RL C3KO and WT muscles (Supplementary Material, Table S1). Transcription of many heat shock genes is controlled by heat shock factor 1 (HSF-1) (36). Under our experimental conditions, HSF-1 was upregulated in WT but not in C3KO RL muscle (Fig. 6B and C). In parallel, expression of HSP90, a component of the HSF-1 protein complex and a transcriptional target of HSF-1, was also reduced in C3KO muscles compared with WT muscles (Fig. 6B and C).

**Figure 7.**
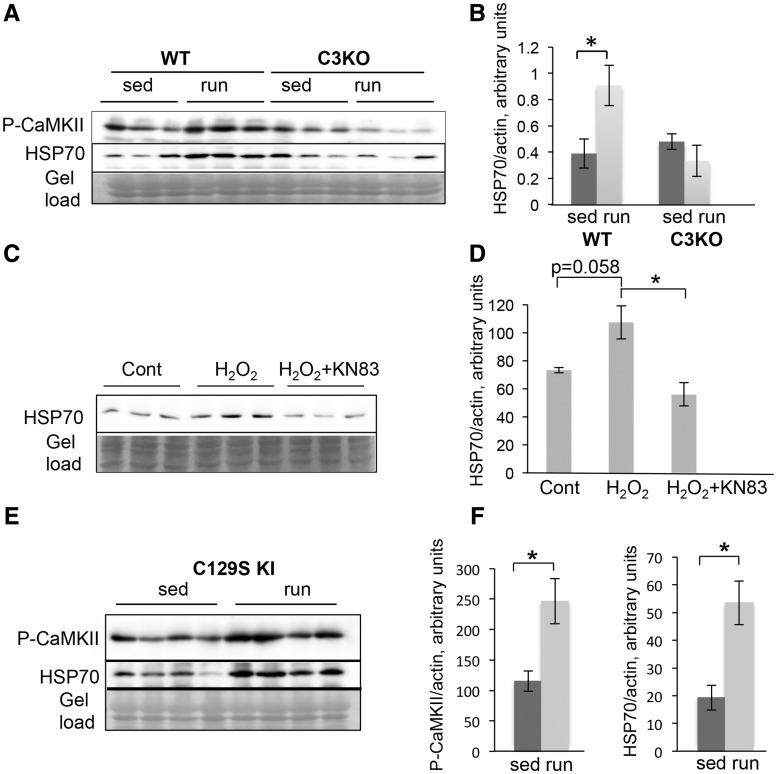
** **Levels of HSP70 correlated with activation of CaMKII in C2C12 cells and in adult muscle. **(A)** P-CaMKII and HSP70 were upregulated in response endurance exercise in WT but not in C3KO muscles. Western blot analysis of activated P-CaMKII and HSP70 expression in WT and C3KO sedentary control muscles (sed) and muscles subjected to running exercise (run). **(B)** Quantitative analysis of HSP70 western blot shown in (A). **(C)** HSP70 was upregulated in C2C12 myotubes in response to cell stress caused by incubation with 0.5 mM H_2_O_2_; inhibition of CaMKII by 20 μM KN83 completely blocked HSP70 induction. **(D)** Quantitative analysis of the western blot shown in (C). **(E)** Western blot analysis of activated P-CaMKII and HSP70 expression in CAPN3-C129S KI mice subjected to running exercise. **(F)** Quantitative analysis of western blots shown in (E). Asterisks indicate statistical significance with *P* < 0.05 considered statistically significant. Vertical bars represent SD, *n* = 3 for each group.

HSP70 expression and secretion has also been shown to be induced in response to endurance exercise (32). Previously, we found that C3KO muscles were not able to fully activate the proper program of gene expression in response to this type of exercise (8), thus we sought to investigate whether HSP70 induction occurred normally in C3KO muscles in this context. As shown in Figure 7A and B, HSP70 was increased in WT exercised muscles but was not increased in C3KO muscles. Taken together, these data suggest that the CAPN3/CAMKII axis is important for activation of stress signals necessary for muscle remodeling and adaptation.

### CaMKII activation controls HSP70 induction in response to stress conditions

It is well known that multiple signaling pathways can control induction of HSPs. In cardiomyocytes, CaMKIIδ regulates the stress response through expression of inducible HSP70 (37). Our data also demonstrate a correlation between activated CaMKIIβ and the expression of HSP70 in RL muscles (Figs 1B and 7A). Based on these observations, we hypothesized that CaMKIIβ may also be involved in regulating cell stress response, including expression of HSP70, in skeletal muscle cells.

To directly test the hypothesis that CaMKIIβ signaling activates HSP70 expression in response to cellular stress in a simple model system, we used C2C12 muscle cells. We first analyzed the expression of different forms of CaMKII in these cells during myogenic differentiation. As shown in Supplementary Material, Figure S4A and B, CaMKIIδ is the only form of CaMKII that is expressed in myoblasts, but during the course of differentiation, CAMKIIδ decreases significantly and CaMKIIβ and CaMKIIγ become the major forms present in myotubes. Levels of HSP70 remain the same during myogenic differentiation (Supplementary Material, Fig. S4C). Based on these observations we chose to conduct further experiments with fully differentiated myotubes, because they are more representative of muscle fibers.

To determine whether CaMKII activation is responsive to cell stress in the C2C12 system, 6-day myotubes were incubated with different concentrations of H_2_O_2._ This treatment induced a cell stress response and CaMKIIδ activation in cardyomyocytes (37). As shown in Supplementary Material, Figure S5, incubation with H_2_O_2_ caused a dose-dependent activation of CaMKβ and induced expression of HSP70 in C2C12 myotubes. Moreover, we showed the HSP70 induction is specific to CaMKII activation, since inhibition of CaMKII activity with KN93 completely abolished the HSP70 induction (Fig. 7C and D). These results show that CaMKII is involved in regulation of HSP70 expression in response to stress conditions in skeletal muscle cells.

We next sought to determine whether restoring CaMKII activation *in vivo* would also reestablish HSP70 induction in response to exercise. Our studies showed that HSP70 is similarly induced by exercise or muscle reloading (Figs 6 and 7); thus, we chose to use treadmill running to test this question (as a less stressful procedure than HS/RL). Previous data suggest that CAPN3 helps to maintain the structural integrity of the triad protein complex necessary for normal Ca^2+^ release (12,14). To address whether restoration of triad integrity and Ca^2+^ release would restore CaMKIIβ activation and HSP70 levels, we used a knock-in (KI) mouse model generated by Ojima *et al.* (13), in which the WT *Capn3* gene was replaced with its proteolytically inactive C129S mutant (C129S KI). These mice possess some pathological features of calpainopathy, however, structural integrity of the triad and Ca^2+^ release upon activation were completely restored compared to C3KO mice (12–14). To test whether restoration of the triad and Ca^2+^ would normalize CaMKIIβ and HSP70 levels, we subjected C129S KI mice to running exercise and assessed activation of CaMKIIβ by western blotting. We found that CaMKIIβ activation was indeed normalized when structurally intact CAPN3 was restored. Accordingly, the induction of HSP70 in response to running exercise was also restored (Fig. 7E and F). Taken together these data suggest that the Capn3 and CaMKII-mediated signaling regulates expression of inducible HSP70 in response to physiological stress in skeletal muscle cells.

## Discussion

Skeletal muscle has the capacity to remodel in response to different types of muscle activity, oxygen changes, nutrient availability and hormonal status (38). The ability of muscle to sense and distinguish these cues is crucial for its adaptive response, i.e. activation of a specific set of signaling pathways that regulate gene expression and ultimately influence its phenotype. We previously showed that CAPN3/CaMKIIβ signaling is one such pathway that is activated after running exercise and this signaling is blunted in C3KO muscles, thus compromising their ability to adapt and switch to a more oxidative phenotype (8,9). These results led to the hypothesis that the triad protein complex, that includes CAPN3, and CaMKIIβ may act as a sensor of environmental cues that trigger muscle adaptation.

To determine the extent to which CaMKII-mediated signaling is involved as a global trigger of adaptation and remodeling, we utilized HS/RL to elicit a different type of muscle adaptation. In this study, we demonstrate that CaMKIIβ is similarly activated during HS/RL, and showed that this pathway is attenuated in CAPN3 deficient muscles. Thus, CAPN3–CaMKIIβ-mediated signaling appears to orchestrate muscle remodeling in at least two conditions that trigger muscle remodeling. However, we observed that gene expression patterns differ, suggesting that downstream targets of CAPN3/CaMKII-signaling fluctuate depending on the type of loading that the muscle experiences. These results suggest that CaMKII is a nodal point that is fine tuned by downstream signals, to modulate the adaptive response to match the type of loading cues.

In these studies, we showed that RL induces activation of cell stress response genes and that this process is attenuated in CAPN3 deficient muscles. For example, expression of inducible HSP70 is not activated in C3KO muscles compared to WT muscles. Recent studies using genetic ablation and overexpression of HSP70 have provided significant experimental evidence that HSP70 is indispensable in muscle adaptation and recovery after injury. Hsp70 transgenic mice are less susceptible to muscle injury following lengthening muscle contractions and these mice also showed improved muscle recovery (39). In a cryolesioning injury model, injection of an HSP70-expressing plasmid 3 days post-injury was shown to enhance muscle regeneration (40), thus demonstrating that increased HSP70 not only protects against muscle damage but also intensifies the muscle recovery process. HSP70 KO mice subjected to cardiotoxin-induced muscle injury showed a delayed inflammatory response that was associated with increased fibrosis and decreased muscle regeneration and fiber growth. Injection of recombinant HSP70 to HSP70 KO muscle at the time of injury was shown to restore normal immune cell infiltration to the site of injury (32,35). The exact mechanisms of how HSP70 regulates the immune response still remain to be elucidated. It is possible that secreted HSP70 may act as a DAMP through direct interaction with immune cells to facilitate the immune response.

Transcription of heat shock genes is controlled by HSFs (41). Among those HSFs, HSF-1 plays a crucial role in inducing expression of *hsp* genes in response to various stress conditions and is expressed at the highest level in skeletal muscles (according to our RNA-seq data). HSF-1 deficiency was shown to delay muscle growth in response to various stimuli including RL after disuse atrophy, compensatory overloading and heat stress (21,22,42). Our data showed that HSF-1 is greatly reduced in C3KO RL muscles (Fig. 6). Activation of the stress response by HSF-1 occurs downstream of multiple signaling pathways including Ca^2+^-CaMKII (37) and our data are consistent with the notion that CaMKII signaling is necessary for induction of HSP70.

In these studies, we observed that the reduction in HSP70 correlates with decreased infiltration of immune cells in C3KO muscles during muscle reloading. Interestingly, while inflammation is a very common feature of many muscular dystrophies, LGMD2A biopsies are distinguished in that most demonstrate an eosinophilia at the earliest stages of the disease (43–45). In contrast, in Duchenne muscular dystrophy, the infiltrate is primarily comprised of monocytes/macrophages and neutrophils (46,47). It is possible that the reduction in these myeloid populations in LGMD2A muscles is due to a compromised ability to initiate a stress response following increased muscle loading or damage.

It is widely accepted that immune cells must infiltrate injured muscle with a particular order and timing in order to achieve successful muscle recovery, as immune cells remove necrotic fiber debris and secrete pro-myogenic factors that control both MPC proliferation as well as terminal myogenic differentiation (28). Our RNA-seq analysis revealed decreased expression of DNA replication and cell proliferation genes, decreased pro-myogenic cytokines, and a concomitant down-regulation of myogenin in C3KO RL muscles compared to WT RL muscles. This observation supports the notion that reduction in myoblast activation may be one of the reasons that C3KO muscles fail to grow following HS/RL. There are conflicting reports in the literature on whether or not activation of MPCs is required for muscle growth after a period of disuse (48). Our data of increased expression of cell proliferation genes and myogenin suggest that activated MPSs contribute to muscle growth or regeneration upon increased loading after atrophy. Taken together, our data support the hypothesis that lack of proper activation of HSP70 in C3KO mice leads to a diminished immune response and compromised myogenesis in response to muscle RL. The question remains whether or not these changes reflect a temporary delay in the C3KO growth response that can be compensated for at later stages of recovery. The fact that C3KO mice are born with the same weight as WT mice, but show decreased post-natal growth and never reach WT muscle mass (6), suggests a deficit in post-natal growth as a primary mechanism underlying calpainopathy.

Taken together, our data support the hypothesis that the triad-associated protein complex (which includes CAPN3 and CaMKIIβ), plays a role in sensing mechanical stress induced by at least two types of muscle loading: running exercise and reloading after atrophy. Our data also reveal that CaMKII signaling induces expression of HSP70 which signals myeloid cell infiltration necessary for normal muscle regeneration. In the absence of CAPN3, immune cell influx is attenuated and expression of pro-myogenic factors is reduced. As a result, cell proliferation and myogenic differentiation is compromised, contributing to a growth deficit under reloading conditions observed in C3KO mice.

Other factors and pathways may also contribute to the inability of C3KO muscle to regrow in response to experimental intervention. Recently, Yalvac *et al.* (49) demonstrated a failure of C3KO muscle to regenerate after repeated cycles of cardiotoxin injections. Muscles were examined after 4 and 12 weeks after the last injection, which is beyond the initial phase of inflammatory and regenerative responses to severe muscle injury. The data suggest that C3KO muscles fail to adjust their metabolism during regeneration and that this maladaptation may be attributable to altered AMPK signaling (49). In line with these observations, our RNA-seq data also demonstrated that the genes involved in oxidative metabolism were downregulated during a period of intense muscle growth (reloaded muscles) in WT mice but failed to undergo similar changes in C3KO muscles (Fig. 2).

Collectively, these data suggest that the inability to activate compensatory adult muscle growth may be responsible for the decreased muscle mass observed in C3KO mice and typical for LGMD2A patients. These studies also accentuate the question of whether or not moderate physical exercise could be beneficial for LGMD2A patients and should be taken into account when considering proper physical therapies for these patients.

## Materials and Methods

### Animal use and HS/RL experiment

All experimental protocols and the use of animals were in accordance with the National Institutes of Health Guide for Care and Use of Laboratory Animals and approved by the UCLA Institutional Animal Care and Use Committee. C57 BL/6 (WT) mice were obtained from the Jackson Laboratories. C3KO mice were previously described in (6) and they are congenic to C57/BL6. C129S KI (C129S KI) mice were generated and described by Dr. Sorimachi’s lab (11).

WT and C3KO male mice (4–5 months of age) were subjected to 10 days of hindlimb unloading by elevating the pelvis, so that the feet of the hindlimb did not contact the cage floor [described in detail in (50)]. One group of mice (suspended, *n* = 6 for each genotype) was sacrificed at the end of the suspension period, while the other group (reloaded, *n* = 6 for each genotype) was allowed to return to the normal cage activity for 4 days, after which time the mice were sacrificed and both solei were carefully dissected from each mouse and weighted.

Running exercise was performed according to the treadmill running protocol described previously in (8). WT, C3KO and C129S KI male mice (4–5 months of age) were used (*n* = 5 per group) for these experiments.

### RNA-seq and data analysis

RNA-sequencing libraries were constructed with the TruSeq RNA Sample Prep Kits v2 from Illumina. RNA from two animals from the same group were combined together to generate 12 RNA samples that were indexed with different adapters and pooled for paired-end 100 bp sequencing on two lanes of Illumina HiSeq2000. RNA-seq reads were aligned with TopHat v2.0.8b (51) to the mouse genome, version mm9. The average TopHat alignment rate was 90.67%, resulting in an average of 28.9 million reads per sample. Transcripts were assessed by Cufflinks v2.1.1 (52), using a GTF file based on Ensemble Mus_musculus_NCBI37. Genes were grouped in custom lists. Heatmaps were created in R, version 2.14.1, log10 of FPKM values are shown.

### Statistical analysis

Differentially expressed genes were found by Cuffdiff, as part of the Cufflinks package (52). Cuffdiff tests the statistical significance of the expression of different groups. This is done by testing the observed log-fold change of the gene’s expression against the null hypothesis of no change. Genes were considered significant if they satisfied a threshold of FDR multiple testing correction value <0.05. WT reloaded samples were compared to WT suspended samples with Cuffdiff. A set of genes that were significantly different between WT reloaded and WT suspended muscles were considered as the normal pattern of changes in gene regulation induced by loading (‘normal gene signature’). These genes were then assessed in the comparison of C3KO reloaded samples to C3KO suspended samples.

For the two group comparisons two-tailed Student’s *t*-test was used. One-way ANOVA was used to calculate statistical significance when comparing multiple pairs. The statistical analysis was performed using IBM SPSS Statistics software. *P*-values < 0.05 were considered statistically significant.

### Real-time PCR validation of RNA-seq data

cDNA was generated using iScript Reverse Transcriptase Supermix (Bio-Rad) and was used for real-time PCR with ITaq Universal SYBR Green Supermix (BioRad) according to manufacturer’s instructions. All real-time PCR reactions were run in CFX Connect Real-Time PCR System (Bio-Rad). Primers for real-time PCR were selected to span intron–exon junctions (when possible) and were first tested in regular PCR amplification to ensure the production of a single band in each case. The following primer pairs were used:

*Itgax* (CD11c)—5’ cgagttgcagaaggccaagt (frw), 5’ aaaaagtcatctgtgagcctcc (rev);

*Itgam* (CD11b)—5’ gtgctcttggctctcatcact (frw), 5’ gggaggtcaatgcatggagaa (rev);

*Ccl2* (MCP-1)—5’ ctgtcatgcttctgggcctg (frw), 5’ cttggtgacaaaaactacagcttc (rev);

*Ccl12* (MCP-5)—5’ cagtcctcaggtattggctgg (frw), 5’ atggtcctgaagatcacagctt (rev);

*Myogenin—*5’ tccagtacattgagcgcctac (frw), 5’*—*gagcaaatgatctcctgggttg (rev);

*Chrnγ—*5’ gctgtctgcctgggggc (frw), 5’ agggcctcctctcgttcatt (rev);

*Hspa1a* (HSP70) 5’—caccatcgaggaggtggattag (frw), 5’ agcccacgtgcaatacacaaag (rev).

### Immunohistochemistry

Frozen solei cross-sections were fixed with cold acetone for 10 min and stained with anti-CD68 antibody (AbD Serotec) followed by secondary anti-rat biotinylated antibody (Vector Laboraories). Staining was visualized using AEC Peroxidase Substrate Kit (Vector Laboratories). Slides were analyzed using AxioVision Software from Zeiss.

### Muscle extract preparation and western blot analysis

For western blot analysis, solei were homogenized in reducing sample buffer (80 mM Tris-HCl, pH6.8, 2% sodium dodecyl sulfate, 10% glycerol and 0.1 M dithiothreitol) supplemented with Halt protease and phosphatase inhibitor cocktail (ThermoFisher Scientific). The following antibodies were used for western blot analysis: anti-HSF-1 (Santa Cruz Biotechnology), anti-hsp70 (StressMarq), anti-hsp90 (BD Transduction Laboratories), anti-P-CaMKII (ThermoFisher Scientific), anti-CaMKIIβ (Invitrogen). The quantitative analysis was performed using ImageJ software.

### Cell culture experiments

C2C12 mouse myogenic cells were grown in Dulbecco Modified Eagle Medium (DMEM) supplemented with 10% fetal bovine serum and antibiotics (all cell culture reagents were from ThermoFisher Scientific). To induce myogenic differentiation, cells were washed with serum-free DMEM and transferred to differentiation medium (DMEM supplemented with 2% horse serum). After 6 days of differentiation, myotubes were incubated overnight in the presence of 0.5 mM H_2_O_2_, with or without CaMKII inhibitor KN93 (20 μM, Sigma). All experiments were done in triplicates.

For western blot analysis cells were collected in PBS using cell scraper. Cell pellets were resuspended in reducing sample buffer (80mM Tris-HCl, pH6.8, 2% sodium dodecyl sulfate, 10% glycerol and 0.1 M dithiothreitol) supplemented with Halt protease and phosphatase inhibitor cocktail (ThermoFisher Scientific).

## Supplementary Material

Supplementary Material is available at *HMG* online.

## Supplementary Material

Supplementary Figures

Supplementary Tables
