# Supplementary material for: Calpain 3 and CaMKIIβ signaling are required to induce HSP70 necessary for adaptive muscle growth after atrophy
Source: Hum Mol Genet. 2018 Mar 8;27(9):1642–53. doi: 10.1093/hmg/ddy071 (PMC5905633; doi:10.1093/hmg/ddy071)
Supplement: Supplementary Figures [file ddy071_suppl_figures.pptx]

## Slide 1
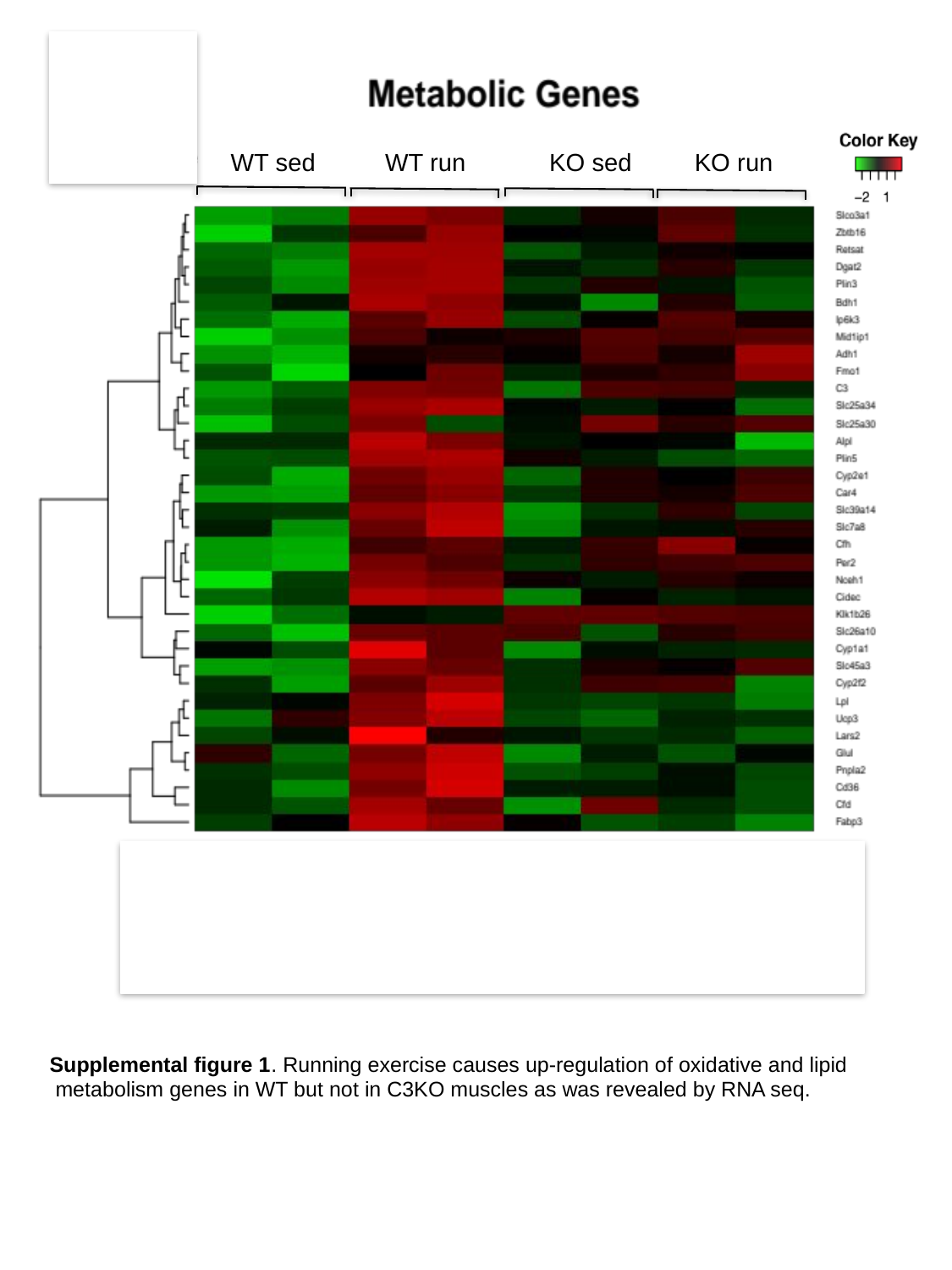

WT sed WT run KO sed KO run
Supplemental figure 1. Running exercise causes up-regulation of oxidative and lipid
 metabolism genes in WT but not in C3KO muscles as was revealed by RNA seq.

## Slide 2
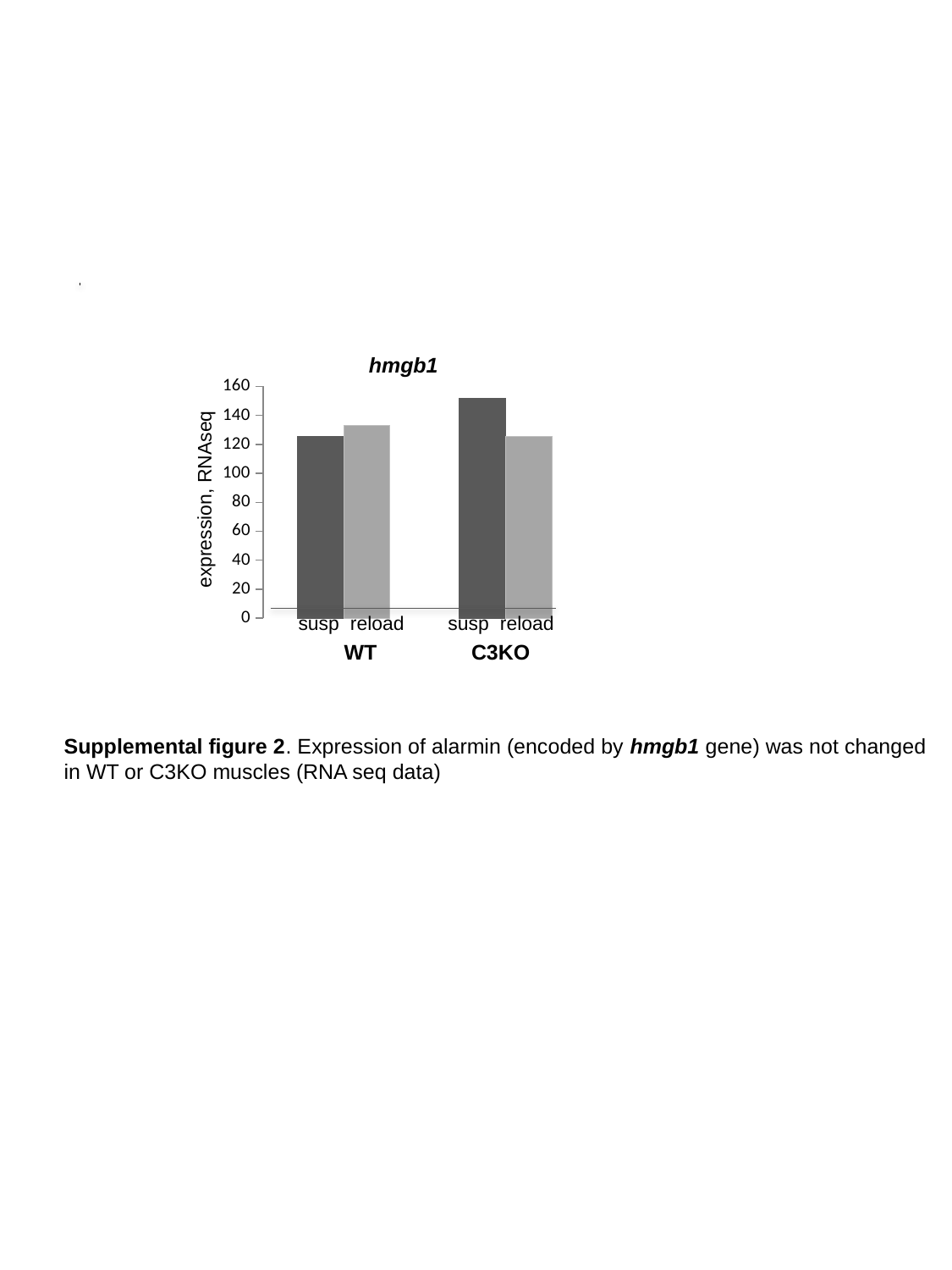

hmgb1
### Chart
| Category | | |
|---|---|---| expression, RNAseq
susp reload susp reload
WT C3KO
Supplemental figure 2. Expression of alarmin (encoded by hmgb1 gene) was not changed
in WT or C3KO muscles (RNA seq data)

## Slide 3
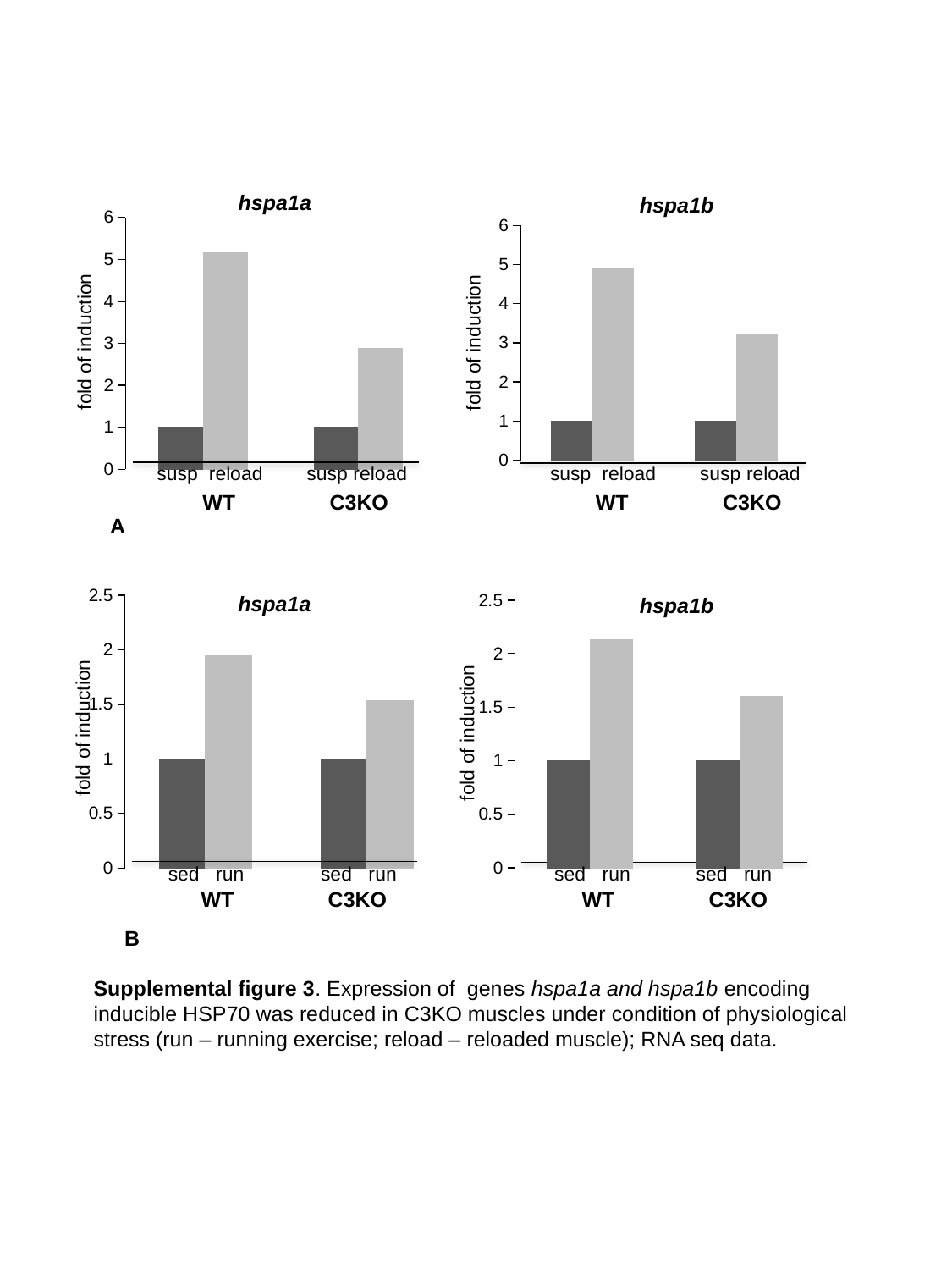

hspa1a
hspa1b
### Chart
| Category | | |
|---|---|---|fold of induction
### Chart
| Category | | |
|---|---|---|fold of induction
susp reload susp reload
susp reload susp reload
WT C3KO
WT C3KO
A
### Chart
| Category | | |
|---|---|---|
### Chart
| Category | | |
|---|---|---|hspa1a
hspa1b
fold of induction
fold of induction
sed run sed run
sed run sed run
WT C3KO
WT C3KO
B
Supplemental figure 3. Expression of genes hspa1a and hspa1b encoding inducible HSP70 was reduced in C3KO muscles under condition of physiological stress (run – running exercise; reload – reloaded muscle); RNA seq data.

## Slide 4
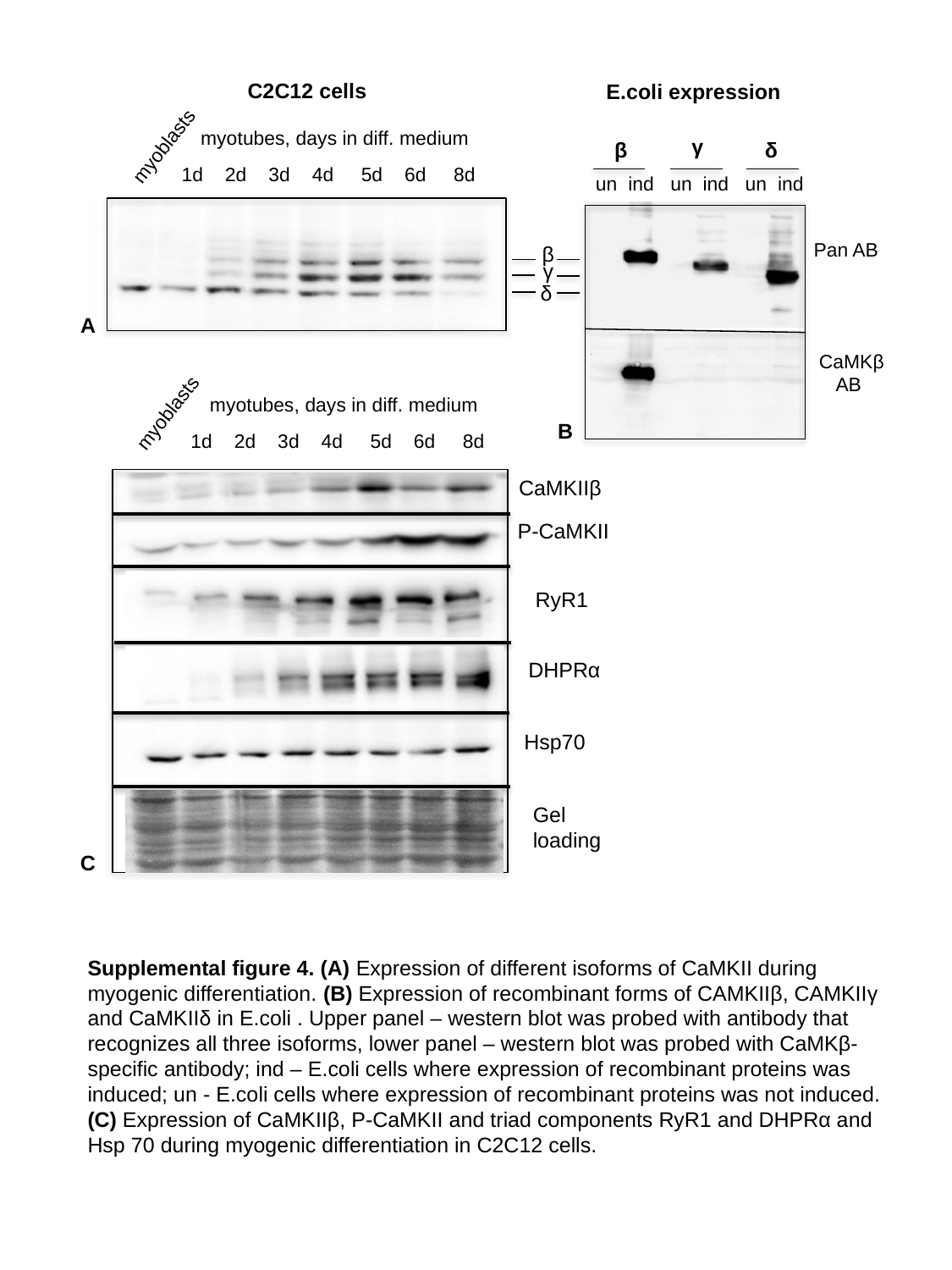

C2C12 cells
E.coli expression
myotubes, days in diff. medium
γ
myoblasts
δ
β
1d 2d 3d 4d 5d 6d 8d
un ind un ind un ind
A
Pan AB
β
γ
δ
 CaMKβ
 AB
myotubes, days in diff. medium
myoblasts
1d 2d 3d 4d 5d 6d 8d
CaMKIIβ
P-CaMKII
RyR1
DHPRα
Hsp70
Gel
loading
B
C
Supplemental figure 4. (A) Expression of different isoforms of CaMKII during
myogenic differentiation. (B) Expression of recombinant forms of CAMKIIβ, CAMKIIγ
and CaMKIIδ in E.coli . Upper panel – western blot was probed with antibody that
recognizes all three isoforms, lower panel – western blot was probed with CaMKβ-specific antibody; ind – E.coli cells where expression of recombinant proteins was induced; un - E.coli cells where expression of recombinant proteins was not induced.
(C) Expression of CaMKIIβ, P-CaMKII and triad components RyR1 and DHPRα and Hsp 70 during myogenic differentiation in C2C12 cells.

## Slide 5
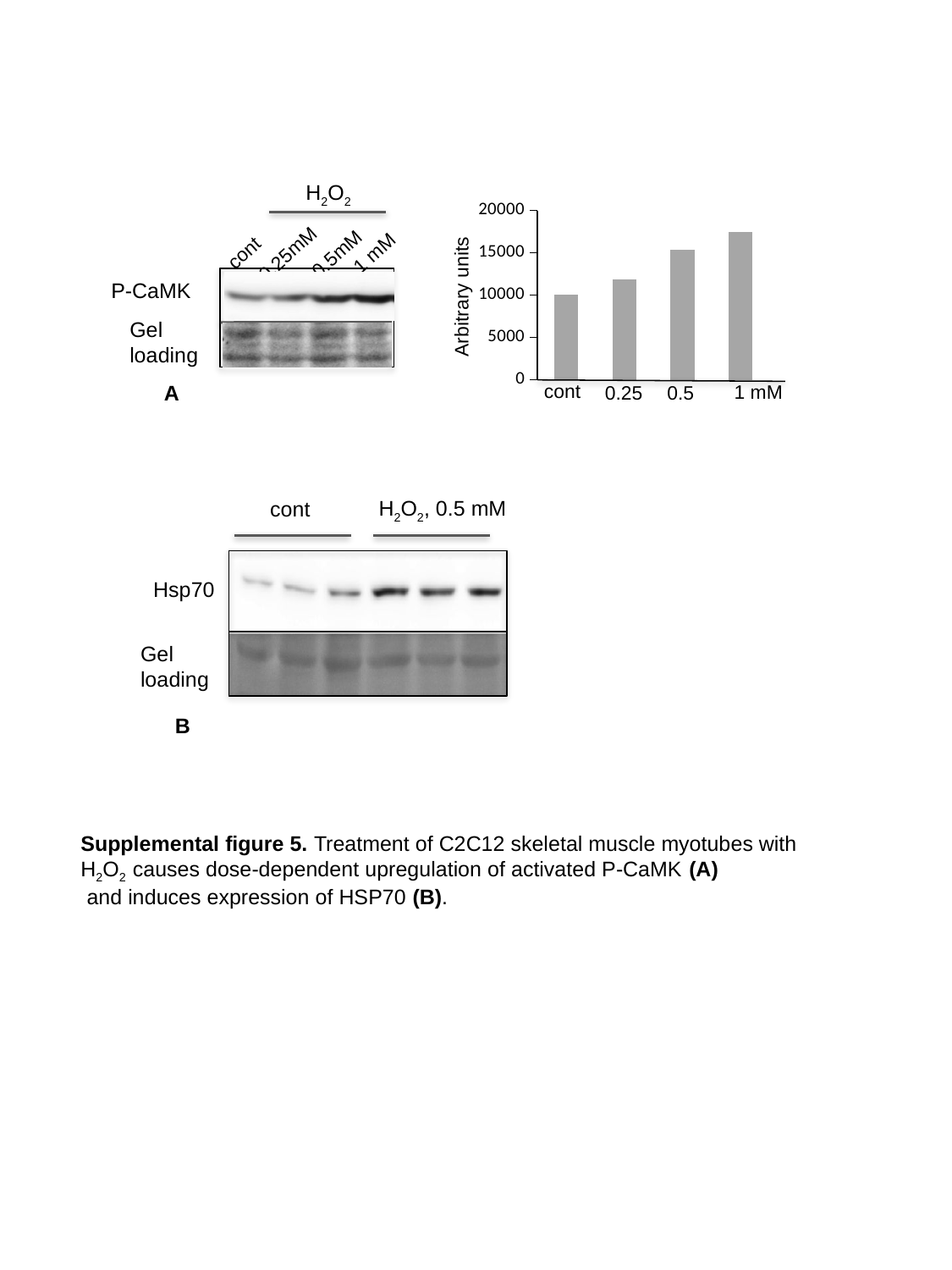

H2O2
cont
1 mM
0.5mM
0.25mM
v
P-CaMK
Gel
loading
A
### Chart
| Category | |
|---|---|Arbitrary units
cont
1 mM
0.5
 0.25
H2O2, 0.5 mM
cont
Hsp70
Gel
loading
B
Supplemental figure 5. Treatment of C2C12 skeletal muscle myotubes with
H2O2 causes dose-dependent upregulation of activated P-CaMK (A)
 and induces expression of HSP70 (B).
