# Supplementary material for: Calpain 3 and CaMKIIβ signaling are required to induce HSP70 necessary for adaptive muscle growth after atrophy
Source: Hum Mol Genet. 2018 Mar 8;27(9):1642–53. doi: 10.1093/hmg/ddy071 (PMC5905633; doi:10.1093/hmg/ddy071)
Supplement: Supplementary Tables [file ddy071_supplemental_tables.docx]

Supplemental table 1. Expression of heat-shock genes

| gene | protein | WT susp | WT reload | KO susp | KO reload | t test, reload |
| --- | --- | --- | --- | --- | --- | --- |
| Dnajb11 | hsp40 | 16.75 | 31.67 | 18.58 | 23.40 | 0.0037 |
| Hsp90b1 | hsp90 family | 33.61 | 84.31 | 32.50 | 48.49 | 0.0007 |
| Trap1 | hsp90 family | 72.65 | 50.74 | 74.24 | 59.04 | 0.0229 |
| Hspb7 | hsp27 | 486.79 | 1709.15 | 503.21 | 1343.35 | 0.0002 |
| Hspa1l | hsp70 family | 2.13 | 6.57 | 2.07 | 5.12 | 0.0625 |
| Hspb9 | alpha-crystallin-related | 0.12 | 0.26 | 0.02 | 0.09 | 0.0145 |
